# Method of estimating the effective zone induced by rapid impact compaction

**DOI:** 10.1038/s41598-021-97912-1

**Published:** 2021-09-15

**Authors:** Shih-Hao Cheng, Shi-Shuenn Chen, Louis Ge

**Affiliations:** 1 Taiwan Building Technology Center, National Taiwan University of Science and Technology, 43, Sec. 4, Keelung Rd., Taipei City, Taiwan, ROC; 2 Department of Civil and Construction Engineering, National Taiwan University of Science and Technology, 43, Sec. 4, Keelung Rd., Taipei City, Taiwan, ROC; 3 Department of Civil Engineering, National Taiwan University, No. 1, Sec. 4, Roosevelt Rd, Taipei, 10617 Taiwan, ROC

**Keywords:** Civil engineering, Imaging techniques, Statistical physics, Environmental impact

## Abstract

This paper proposes a method for estimating the effective zone, including effective depth and effective range of compaction degree, from rapid impact compaction (RIC) on sand layer whose fines content is less than 10%. The proposed method utilizes a string of microelectromechanical system accelerometers to monitor the acceleration at various depths and propagation distances during compaction. To interpret and extract useful information from monitored data, peak-over-threshold (POT) processing and normal distribution function were used to analyze the recorded acceleration. The mean and standard deviation of the threshold peak acceleration were used to evaluate the effective depth and the effective range of compaction degree during RIC compaction. Moreover, spatial contours were used to determine the correlation of the threshold peak acceleration against depth and propagation distance from the RIC impact point. These contours help indicating the distribution of the effect zone after compaction. Lastly, a proposed method is suggested for frequent use in trial tests to quickly determine RIC’s required depth and impact spacing.

## Introduction

Hydraulic filling is an important construction method for land reclamation. However, the stability and material properties of reclaimed lands are affected by factors such as the filled material, particle size distribution, loss of fines and groundwater table. Therefore, a cost-effective and time-efficient soil compaction method is often adopted by engineers for ground improvement to satisfy the required bearing capacity reduce excessive settlement and resist liquefaction. In many cases, the operation of heavy equipment, e.g., the dynamic compaction (DC) method, is restricted to use due to existing structural and geometric constraints in the proximity of a construction site. The rapid impact compaction (RIC) method, an alternate to DC, has excellent machine operability and a high construction rate that highlights its environment-friendly advantages, such as small discrete areas, reduced dust and vibration. The RIC method has been widely applied worldwide as a ground improvement method for cohesionless granular soil or reclaimed lands at medium (< 10 m) compaction depth^1,2^.

Compared to DC, the RIC method has different features, including a considerably smaller impact energy but considerably larger impact rate and the steel compaction foot being constantly in contact with the ground during impact energy transfer^3,4^. Consequently, the effective depths in these two methods differ substantially. However, they have a similar design of performance assessment, mainly evaluating the compaction performance based on the effective depth and compaction degree. These aspects are primarily affected by the site conditions and compaction parameters. The site conditions depend on the groundwater table and soil characteristics, such as the soil type, particle size distribution and fines content. The compaction parameters depend on the compaction foot weight, fall height, blow count, grid spacing and compaction sequence of the selected compactor^2,5^. In addition, all soil compaction methods, including the RIC and DC methods, are experience-oriented according to their design. Civil engineers frequently estimate the compaction parameters to meet the design requirement based on past experience or recorded data for similar soils. To ensure the effectiveness of compaction, trial tests are commonly used to verify and optimize the compaction parameters^6^. However, the empirically determined effective depth and compaction degree are often found not meeting the design requirement^7^. The compaction degree can be enhanced, within its critical effective depth, by reducing the spacing between impact points, increasing the blow count and increasing the total applied energy. Although the effective depth can be increased to certain degree with the applied energy, it is much more dependent on the properties of compacted soil and the groundwater table. Moreover, increasing the effective depth is difficult after the main compaction zone is formed^8^, particularly at sites with an erratic soil condition and a high groundwater table. The said phenomenon also reveals the uniqueness of effective depths caused by different compaction parameters and different sites. Therefore, effective depth must be confirmed through site investigation for each compaction project.

When the site investigation indicates that the trial compaction cannot achieve the required depth or compaction degree, the impact energy, including the foot weight, fall height and blow count must be adjusted until the design requirements are met before a formal construction can be launched. However, this approach leads to a waste of construction time and cost. To accurately evaluate the effective depth during each compaction project, this study conducted RIC trial tests and proposed a monitoring method synchronized with a compaction activity. The proposed method can be performed at any impact point in the test area using a string of microelectromechanical system (MEMS) accelerometers (or called a shape accel array string, SAA string) to simultaneously record the soil particle acceleration induced by impact energy at various depths and various propagation distances under field conditions. Furthermore, normal distribution function was used in the data process and reduction. Specifically, the mean (µ) and the standard deviation (σ) of the threshold peaks from recorded accelerations along various depths were used as indicators to evaluate the effective depth. In addition, being taken out from the recorded accelerations along various propagation distances, they evaluate the effective range of compaction degree for obtaining a reasonable reference for the arrangement of spacing between impact points.

## Background

In general, the RIC method involves using a hydraulic hammer installed on a compactor with a fall height of 1.2–1.5 m and frequency of 30–60 blows/min to impact cohesiveless soils, such as sand, silty sand and gravel. The impact energy is transmitted to the compaction foot (common diameter is approximately 1–2.4 m and weight is 5–16 t) in contact with the ground through the hydraulic hammer and then transferred to the cohesiveless soil. Consequently, the soil particles are rearranged and densified, which leads to soil compaction and an increase in the soil density. Over the past few decades, considerable progress has been made in the RIC technology in terms of the operation experience and functions of the compaction equipment, particularly the positioning system, digital parameter control unit and data acquisition system. These pieces of equipment can be implemented into the RIC compactor to record the working position of the compaction foot and impact performance. Accordingly, RIC technology is regarded as a mature in-situ compaction method.

Further exploration is required on the evaluation of the effective depth after RIC compaction. Some studies^9–12^ have suggested empirical equations based on the energy per blow, enabling effective depth prediction for most in-situ compaction methods. However, Watts and Cooper^6^ noted that the empirical equations suggested by the previous studies are unsuitable for effective depth prediction in the RIC method, which is influenced by the cumulative energy contribution, because these equations did not consider the accumulation of energy and cannot reasonably reflect the variation of soil properties. Oshima and Takada^13^ suggested the use of the total impact energy or total momentum compaction theory for determining the effective depth of the RIC method. Berry et al.^14^ also proposed an empirical rule of using three to four times the diameter of the compaction foot to estimate the effective depth for this method. These approaches have increased the practicality of the effective depth estimation in RIC method. Published case studies involving RIC compaction in-situ are summarized in Table 1, when the compacted soil is heterogeneous and the particle size distribution spreads in a wide range (e.g., granular, miscellaneous, waste or ash fills), the achievable effective depth may vary largely. Table 1 also indicates that the effective depth is reduced when the soil particles are decreasing. Furthermore, the effective depth after RIC compacted in silty sand to gravel sand ranges between 2 and 9 m. Although the variation of soil properties (e.g., the particle size distribution, fines content and soil saturation), groundwater table, and applied impact energy are not indicated in Table 1, the large variation of the effective depth of sand with different particle sizes after RIC compaction highlights the risk of effective depth control due to the uncertainty of site conditions and soil characteristics. Therefore, RIC-related studies have mainly evaluated the performance of adopted compaction parameters on individual sites through site investigation. For example, Watts and Charles^2^, Serridge and Synac^4^, Kristiansen and Davies^16^, Tarawneh et al.^18^, Mohammed et al.^19^, and Vukadin^20^ used trial tests to evaluate the compaction performance of different soils. Their adopted methods involved both invasive and non-invasive site investigation techniques, including the standard penetration test (SPT), the cone penetration test (CPT), the dynamic CPT, the plate bearing test and continuous surface wave (CSW) measurement, to determine the effective depth and compaction degree. These previous studies have contributed to the advancement of RIC, but unfortunately, the past data were obtained at each particular test site and they were not guaranteed to be completely applicable to different sites even if they have similar soil properties.

**Table 1:** Effective depths for the RIC compaction obtained in different studies.

Soil type	References	Effective depths of compaction (m)
Granular fills	Watts and Charles^2^	3
	Building Research Establishment^15^	4
	Serridge and Synac^4^	3–4
	Watts and Cooper^6^	6
Gravel sand	Kristiansen and Davies^16^	6–9
	Berry and Narendranathan^17^	> 6
Sand	Berry et al.^14^	7
	Adam and Paulmich^3^	6
	Watts and Cooper^6^	> 6
	Tarawneh et al.^18^	3–4
Silty sands	Adam and Paulmichl^3^	3.5–4.5
	Building Research Establishment^15^	2–3
Miscellaneous fills or waste fills or Ash fills	Building Research Establishment^15^	4
	Adam and Paulmich^3^	3–5
	Watts and Cooper^6^	1.8–7

In addition to the compaction performance, Thilakasiri et al.^21^, Parvizi and Merrifield^22^, and Parvizi^23^ performed centrifuge model tests to explore the behaviors of excess pore water pressure, wave propagation, and the stress–strain relationship for sand and organic soil at different relative densities under impact load. Ghanbari and Hamidi^24^ and Allouzi et al.^25^ presented the finite element simulation for effective depth prediction and proposed a new evaluation method for determining the optimal blow counts required to meet the ground improvement requirements. Overall, these studies focused on verifying the rationality of the RIC compaction parameters but did not conduct advanced quality control and quality assurance method of the effective zone on site (including effective depth and suitable spacing between impact points).

## Measurement of the particle acceleration caused by soil distortion due to RIC

**Figure 1:**
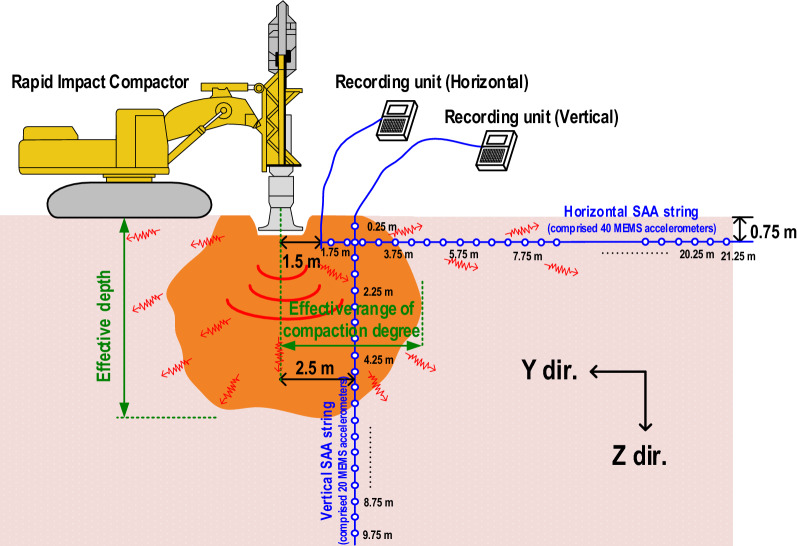
Proposed method for monitoring the soil acceleration caused by shear distortion during RIC compaction at various depths and propagation distances under field conditions (in Y–Z profile).

When compaction energy is effectively applied to the ground, the compression wave (P wave) and shear wave (S wave) interact with soil producing compressions, vibrations, and distortions while increasing the soil density; thus, the larger the soil distortion and soil particle vibration, the greater the densification action on the subsoil^26^. However, the distortion properties of general soil are often not as sensitive as the vibration response of soil particles. Therefore, this study evaluates the effective zone that used SAA string to monitor the particle acceleration induced by the soil shear distortion at various depths and propagation distances during the RIC. To ensure the suitability of the proposed method, this study assumed that the interaction of impact points could only improve the compaction degree within the critical effective depth but could not increase the effective depth. This assumption was supported by the fact that the groundwater is nearly incompressible. When the RIC foot impact ground, it is necessary to synchronize the SAA string to monitor the soil particle accelerations at any impact point selected from the trial test area. As shown in Fig. 1, two SAA strings were installed vertically (2.5 m from the impact point) and horizontally (1.5 m from the impact point) in the ground. The vertical SAA string comprised 20 MEMS accelerometers, which were installed into the ground at an interval of 0.5 m over a depth of 0.25–9.75 m. This string was mainly used to record the particle acceleration response along the soil depth. The horizontal SAA string with 40 MEMS accelerometers was embedded in a 0.75 m excavated trench. These accelerometers were placed at 0.5 m intervals to record the particle acceleration response at a distance of 1.75–21.25 m along the horizontal propagation distance.

**Figure 2:**
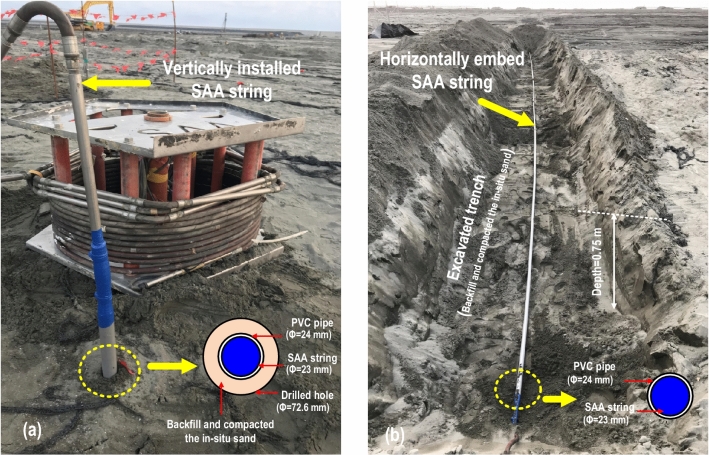
Photos of SAA string measurement on site: (**a**) vertically installed string for monitoring the acceleration caused by compaction at various depths and (**b**) horizontally embedded string for monitoring the acceleration caused by compaction at various propagation distances.

The SAA string is a rope-like rigid sensor array segment with flexible and watertight joints constructed from a hydraulic hose. Each rigid sensor has a length of 0.5 m and contains three MEMS accelerometers that measure the accelerations in the X-direction, Y-direction, and Z-direction at specific locations. In this study, the acceleration response in the Z direction mainly generated by the P wave during RIC compaction, which might be disturbed by the incompressible characteristics of groundwater and soil particles. According to Richart et al.^27^, the wave energy transmitted from the source of footing compaction, the intensity of S wave is stronger than the P wave. Therefore, it can be assumed that the soil shear distortion is mainly caused by the S wave without estimating the acceleration in the Z direction caused by the P wave. As reported by Bennett et al.^28^, real–time monitoring of infrastructure and geotechnical facilities can be exercised by SAA string. Figure 2 displays photos of the on-site SAA string installed or embedded in the ground. In this study, the SAA string was specifically designed for repeated installation or embedding to measure the ground vibration (or soil particle acceleration) during the RIC process. Therefore, the SAA string which had a diameter of 23 mm was protected by a polyvinyl chloride (PVC) pipe with an inner diameter of 24 mm and outer diameter of 32 mm. Although the SAA string and PVC pipe are almost laminated to ensure the accuracy of the recorded data, the zone between the PVC pipe and the drilled hole (or the excavated trench) was backfilled and compacted with in-situ sand to ensure that the acceleration response of the RIC compaction could be effectively captured. Moreover, to obtain the low-strain soil acceleration response effectively, the sensing range of all the MEMS accelerometers in the SAA string was set as 0–1000 Hz, whereas the sampling rate was set as 40 Hz (∆t = 0.025 s).

## Full-scale field test

A hydraulic reclaimed land in southern Taiwan was used for field testing. The dynamic compaction (DC) was selected as the main ground improvement method in the design stage. To demonstrate the suitability of the proposed monitoring method for the effective depth and spacing between compaction points evaluation, this study used the low-impact-energy RIC for conducting a trial test adjacent to the DC working zone. The subsoil conditions of the trial test, compaction sequence and grid pattern, and monitored acceleration records are described as follows.

### Subsoil conditions

**Figure 3:**
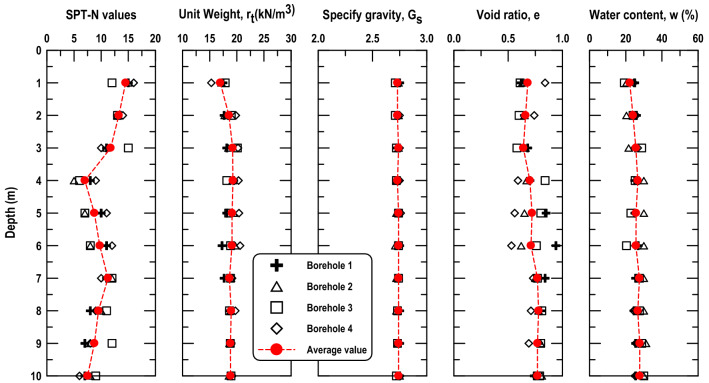
Soil properties of the RIC trial test site.

**Figure 4:**
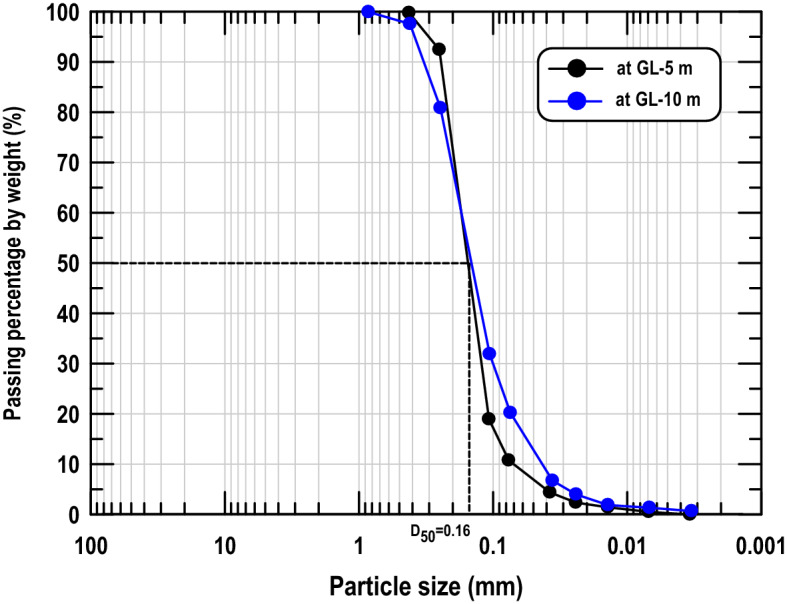
Typical particle size distribution in the RIC trial test site.

The test field is a typical hydraulic fill reclaimed land, whose main filling material is seabed sediments. According to the soil classification system used by the American Association of State Highway and Transportation Officials (AASHTO), the filled soil can be categorized as fine sand. The SPT and the conventional soil tests were conducted on samples collected from four in situ boreholes, the results of which are shown in Fig. 3. It is indicated that the geology of the site within 10 m below the ground surface was almost homogeneous, the mean standard penetration test blow count (SPT-N) was 5–15, the mean unit weight (γ_t_) was approximately 16–20 kN/m^3^, the mean specific gravity (G_s_) was approximately 2.75, the mean void ratio (e) was approximately 0.65–0.9, and the mean water content (ω) was approximately 20–30%. The properties of soil particle size distribution and fines content were crucial factors that affected the soil compaction effectiveness. The particle size distribution in the compaction zone located 5 and 10 m below the ground surface revealed that the average particle size of the soil (D_50_) was approximately 0.16 mm and that the fines content (< 0.075 mm) was less than 10% (Fig. 4). Thus, the soil in the test field was sand with a minimal fines content that was suitable for soil compaction.

**Figure 5:**
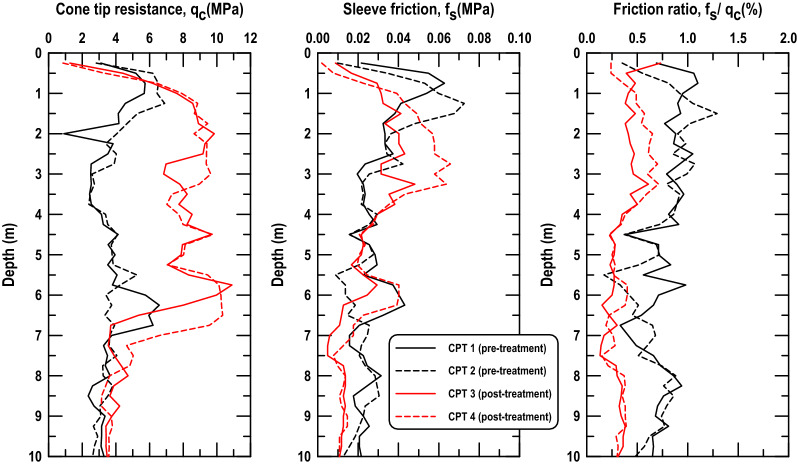
CPT investigation profiles before and after RIC treatment at the trial test site.

The groundwater table of the reclaimed land at the time of trial test was close to 3 m below the ground surface; however, the groundwater table may vary by ± 1 m due to the tidal fluctuation near the port area. Based on the groundwater table and subsoil conditions of the reclaimed land, the design specification of DC compaction indicated that the reclaimed land had high liquefaction potential (liquefaction potential index, PL > 15). Therefore, to evaluate the effectiveness of RIC against liquefaction after compaction, a two-hole CPT test (CPT 1 and CPT 2) was conducted in the trial test area before compaction. The investigation results shown that the average cone tip resistance (q_c_), average sleeve friction (f_s_), and average friction ratio (f_s_/q_c_) were approximately 4 MPa, 0.5–0.6 MPa, and 0.5, respectively (Fig. 5). Furthermore, the liquefaction resistance of the test area after RIC was obtained using the CPT-based evaluation procedure recommended by Robertson^29^ for determining the soil liquefaction potential. According to Robertson^29^, the average q_c_ within a depth of 10 m should be > 8 MPa.

### RIC sequence and grid pattern

**Figure 6:**
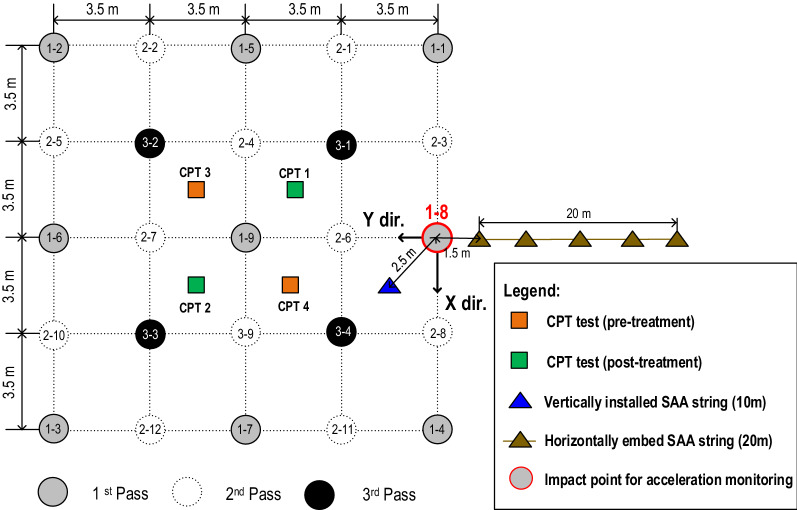
Locations of the CPT investigation test, SAA string measurement, and monitoring impact point as well as the layout of the RIC sequence and grid pattern (in X–Y plane).

The area of the trial test was 14 m × 14 m, and the impact points of RIC were arranged in a grid pattern (Fig. 6). To focus on the suitability of the proposed monitoring method, this study did not examine in detail the effect of various grid spacing and improvement rates but considered the field conditions and the previous studies^30,31^ to arrange the layout of RIC sequence and grid pattern. Compaction was conducted in three passes with a grid spacing of 3.5 m. The detailed compaction sequence and grid layout are displayed in Fig. 6. Information related to the RIC equipment and impact energy is presented in Table 2. Each impact point was produced by impacting a 14 t round hammer 50 times on the ground over a fall height of 1.2 m. The total input energy for each impact point was 840 t-m. Meanwhile, when the depth of compaction foot penetration was 0.9 m, the impact pit was backfilled with the in-situ soil to an initial elevation and compacted to the designed blow counts (50 blow counts) before moving to the next impact point.

**Table 2:** RIC equipment and compaction information.

Specification	Quantity
Foot weight	14 t (the diameter of round hammer = 1.5 m)
Fall height	1.2 m
Blow count	50 blows/ per compaction point
Depth of foot penetration	0.9 m
Fall energy per blow	16.8 t-m
Delivered energy per impact point	840 t-m

To verify the effectiveness of RIC, two CPT tests were conducted at the center of the trial test area 7 days after the three-pass compaction procedure was completed. The CPT results (CPT3 and CPT 4) are displayed in Fig. 5. The q_c_ from post-treatment along the depth to 6–7 m was close to the designed requirement of 8 MPa. This study determined that under the adopted grid pattern, a design effective depth of 10 m could not be achieved with an effective depth of approximately 6–7 m. The result is consistent with the effective depth reported by Adam and Paulmich^3^, Watts and Cooper^6^, Berry et al.^14^ in the sand layer (Table 1).

### Monitored acceleration recorded

**Figure 7:**
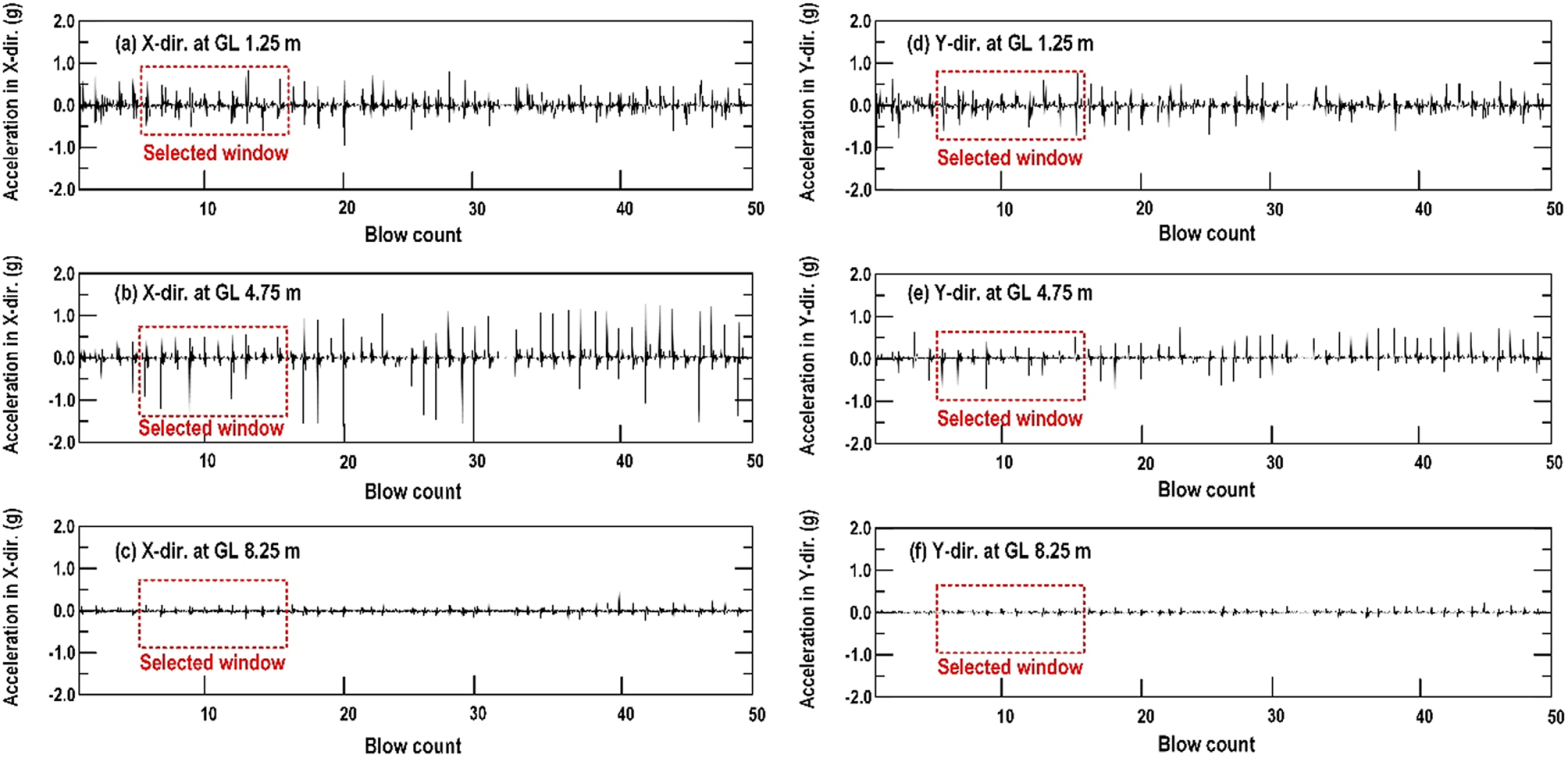
Soil particle acceleration recorded from the vertically installed SAA string during RIC at impact points 1–8: (**a**–**c**) X-direction response at GL values of 1.25, 4.75, and 8.25 m; (**d**–**f**) Y-direction response at GL values of 1.25, 4.75, and 8.25 m (GL, ground level).

**Figure 8:**
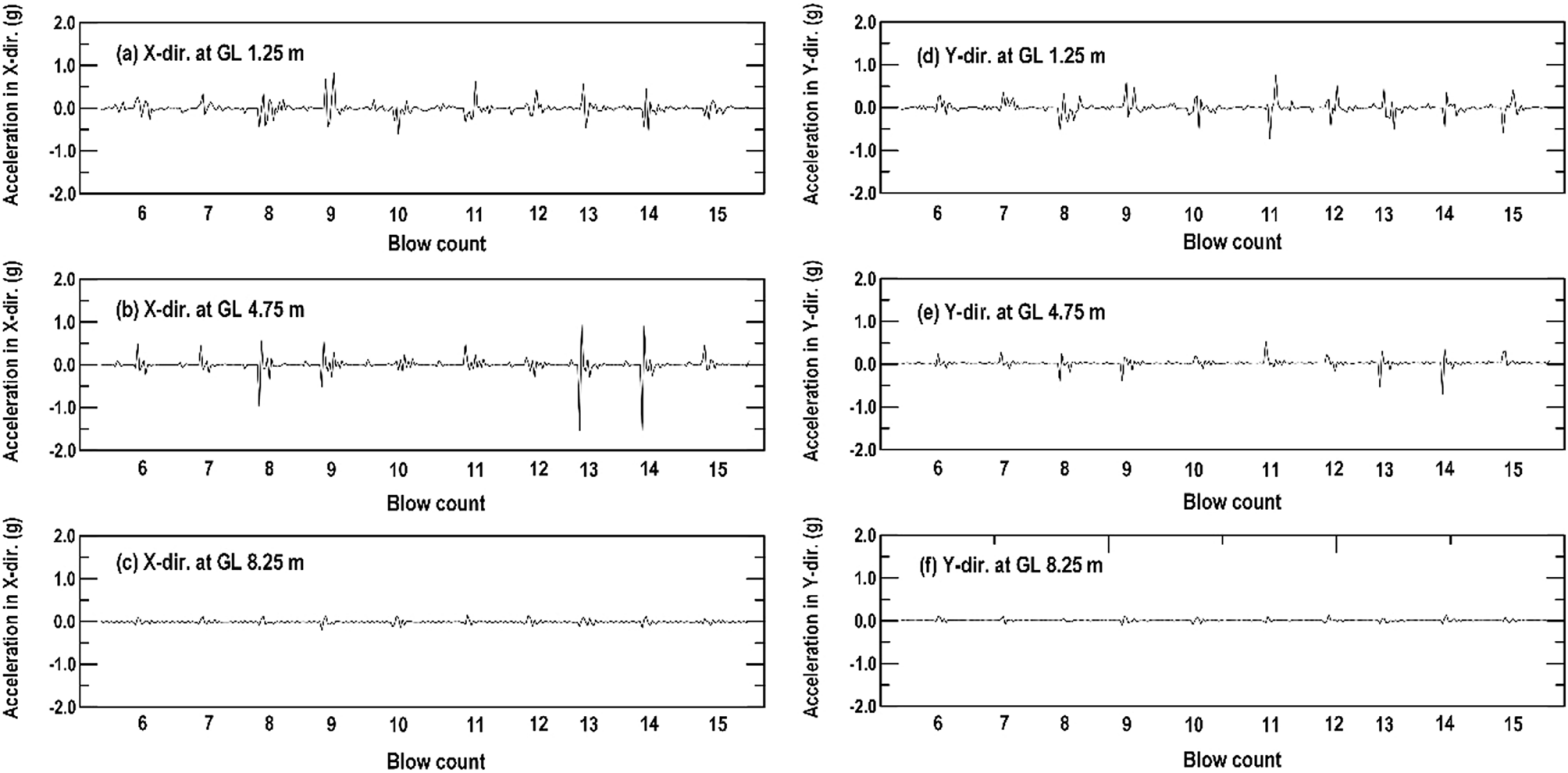
Acceleration record for the selected window in Fig. 7 (impact from 6 to 15 blows): (**a**–**c**) X-direction response at GL values of 1.25, 4.75, and 8.25 m; (**d**–**f**) Y-direction response at GL values of 1.25, 4.75, and 8.25 m (GL, ground level).

As displayed in Figs. 1, 6 and mentioned above, that monitored points can be selected from the trial test area. Two SAA strings were installed vertically (with 20 accelerometers) and embedded horizontally (with 40 accelerometers) around 1–8 impact point. During the RIC process, all the accelerometers simultaneously detected the acceleration generated by the soil shear distortion at the monitoring position. Consider the vertically installed SAA string and the specific depths of 1.25 m, 4.75 m, and 8.25 m as an example. Figure 7 displays the acceleration record due to soil shear distortion in the X-direction (Fig. 7a–c) and Y-direction (Fig. 7d–f) at each blow. The transient waveforms and peak amplitudes for each blow can be clearly observed in the acceleration records of the selected window in Fig. 8 (taken from 6 to 15 blows). However, the real-time acceleration recorded during the RIC process was a signal of random vibration, and useful information was difficult to obtain without data processing. Nevertheless, according to the recorded accelerations at depths of 1.25 m, 4.75 m, and 8.25 m, it indicates that a larger soil distortion may result in larger peak amplitude. Moreover, the soil distortion may enlarge to a critical depth and decreased with increasing depth. Thus, the characteristics of the peak acceleration distribution can be captured at each recording position to evaluate the effective depth of compaction. The distributions of the peak acceleration captured from horizontally embedded SAA string may decrease as the propagation distances increase. It can be used to evaluate the suitable spacing between impact points.

## Data processing and interpretation

When monitoring work is performed at a soil compaction site, the recorded data may contain white noise to varying degrees due to the uneven soil shear distortion (interference of large gravel) or changes in the working direction of the compaction equipment. Because the subsoil at the trial test was almost uniformly distributed, the effect of white noise was non-significant in this study. In addition, in-situ compaction may cause the interruption of monitoring signals due to expected or unexpected conditions (e.g., backfilling of impact pits and moving of machinery), and these unusable signals should also be eliminated. Hence, this study adopted the peak-over-threshold (POT) method to threshold peak accelerations from the vertical SAA string recorded (the response along different depths) and the horizontal SAA string recorded (the response along different propagation distances). Moreover, normal distribution function was used to determine the dispersion degree of the captured peak acceleration at each recorded position. The details are described as follows.

### Distribution of the threshold peak acceleration

**Figure 9:**
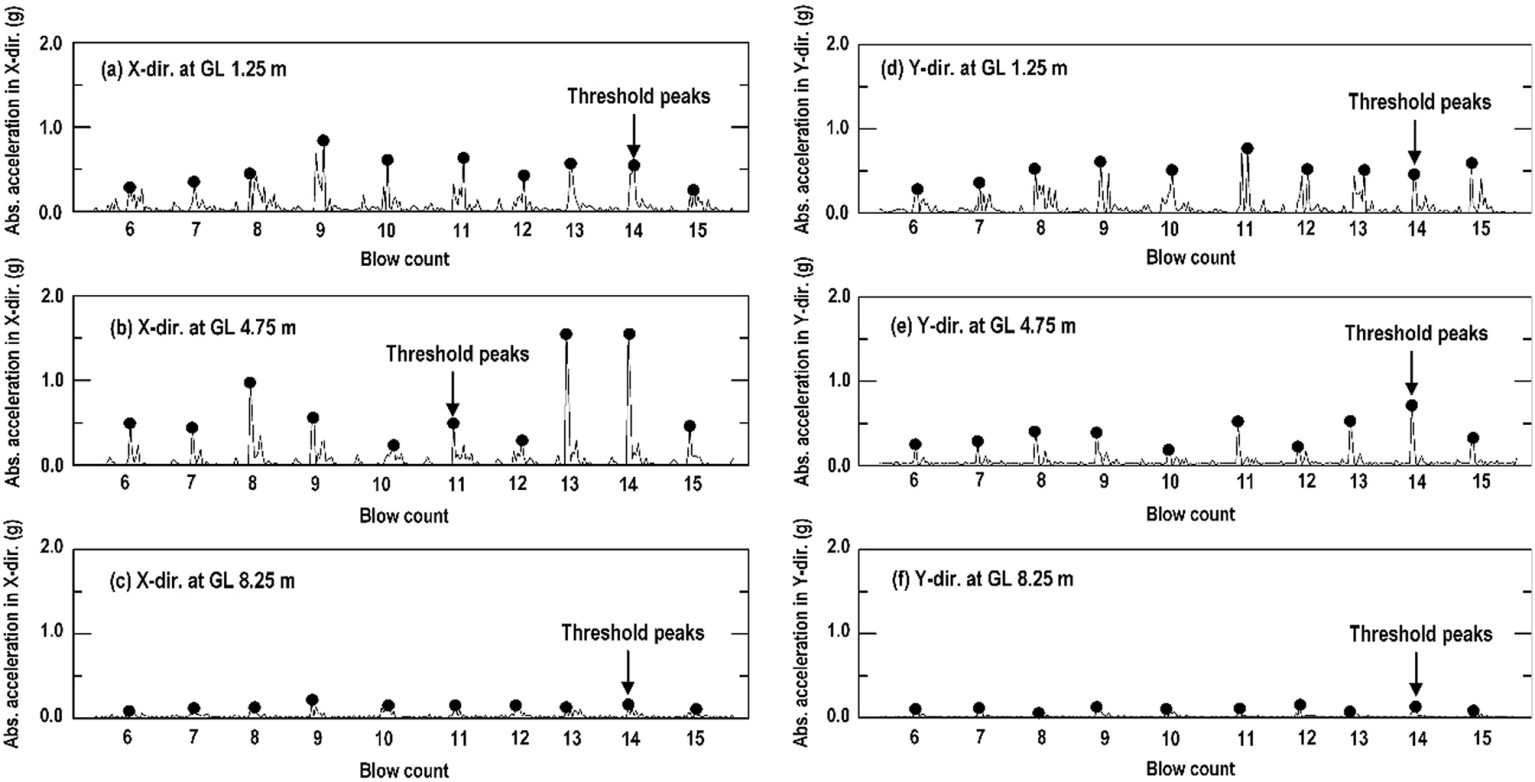
Partial acceleration record and action of POT processing result: (**a**–**c**) X-direction response at GL values of 1.25, 4.75, and 8.25 m; (**d**–**f**) Y-direction response at GL values of 1.25, 4.75, and 8.25 m (GL, ground level).

In time-domain signal processing and feature extraction, peaks, amplitudes, and means are often used as essential indicators for signal interpretation. Therefore, this study took monitored data from Fig. 8 as an example and adopted the POT method to threshold the peak acceleration. Figure 9 illustrates the POT processing result, after eliminating the unusable records and ignoring whether the vibration direction was positive or negative, acceleration is used as the absolute value of the signal to calculate the mean. Moreover, the maximum amplitude of each blow that exceeded the mean was threshold as the peak acceleration.

**Figure 10:**
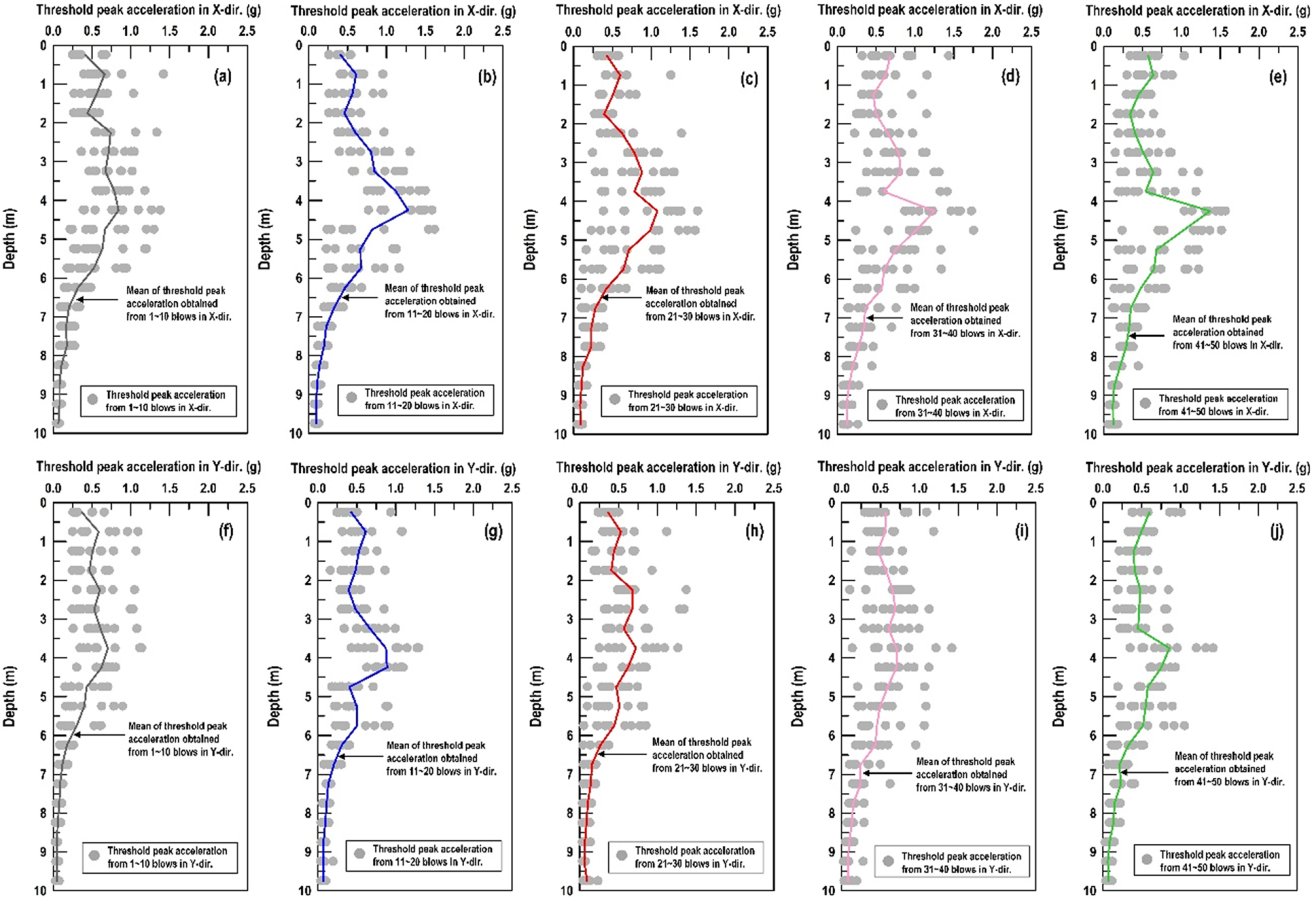
Distributions of the threshold peak acceleration and mean obtained from the vertically installed SAA string during RIC compaction at impact points 1–8: (**a**–**e**) X-direction response during 1–10, 11–20, 21–30, 31–40, and 41–50 blows; (**f**–**j**) Y-direction response during 1–10, 11–20, 21–30, 31–40, and 41–50 blows.

**Figure 11:**
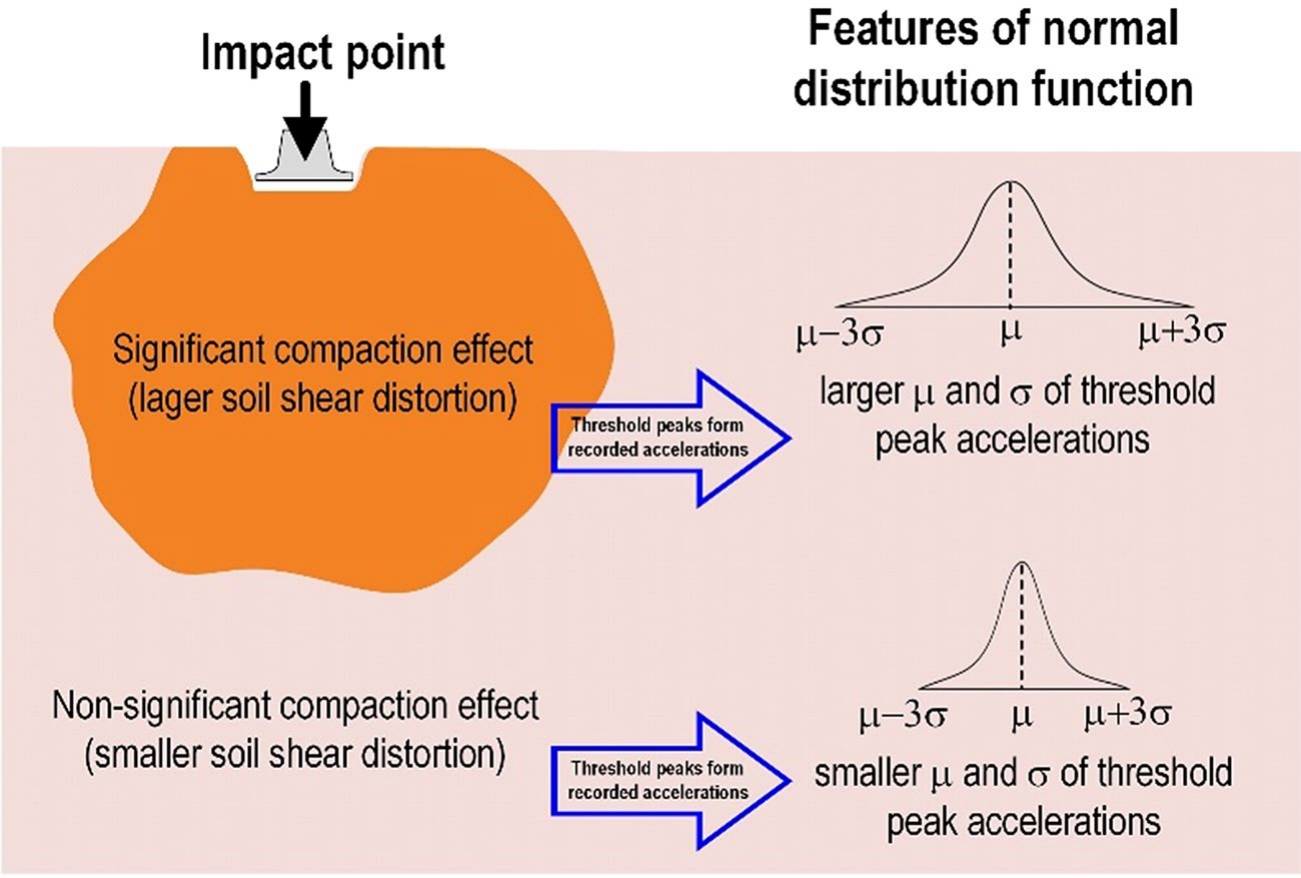
Schematic to illustrate the concept of normal distribution function to represent the threshold peaks features.

**Figure 12:**
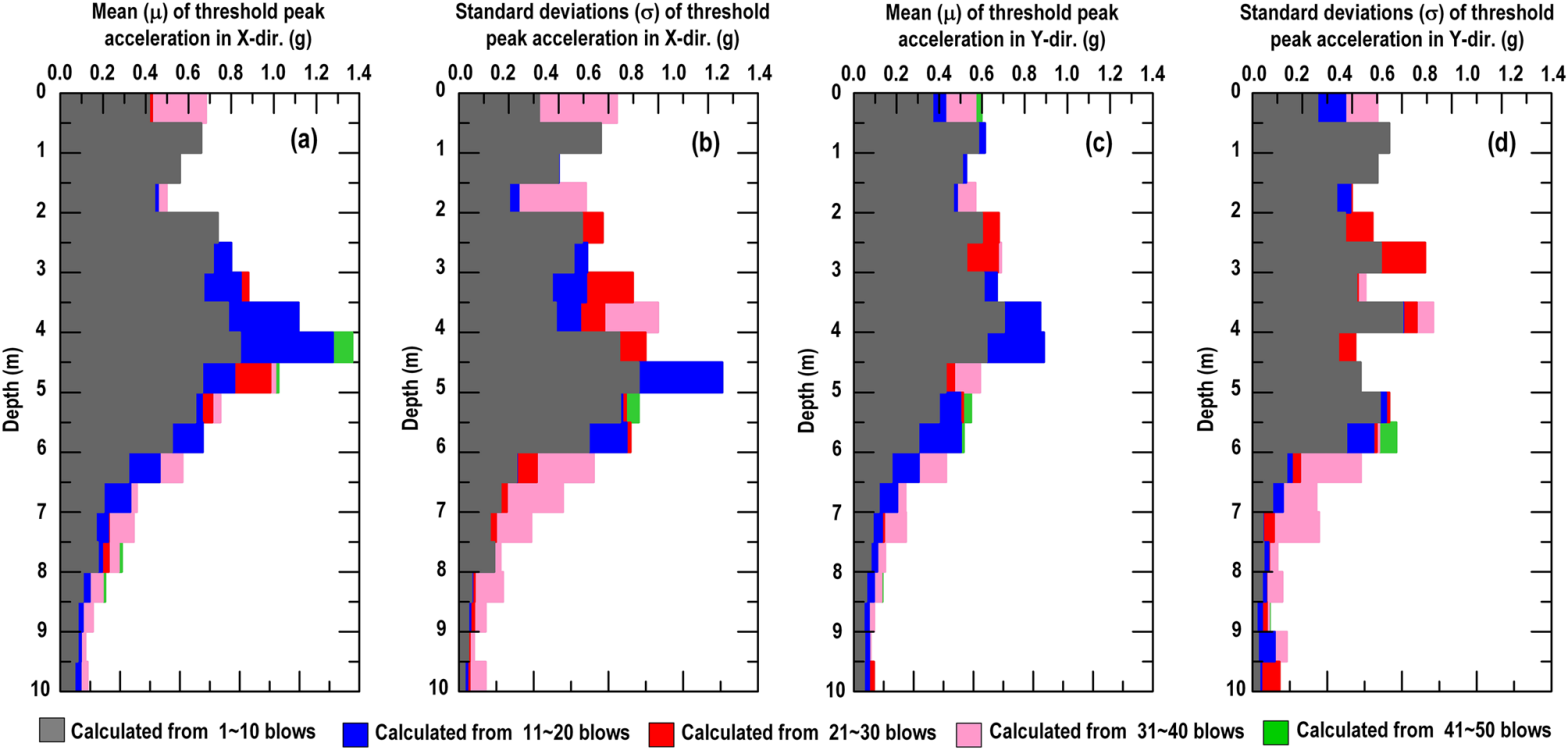
Calculated normal distributions results from the vertically installed SAA string recorded: (**a**, **c**) mean of threshold peak acceleration in X-direction and Y-direction; (**b**, **d**) standard deviation of threshold peak acceleration in X-direction and Y-direction.

Similar procedure as above, Fig. 10 shows that both the X-direction and Y-direction threshold peak acceleration distribution extracted from the vertically installed SAA string recorded. It can be found that the peak acceleration distribution in X-direction (Fig. 10a–e) and Y-direction (Fig. 10f–j) have the same trend. The peak acceleration is enlarged to a certain depth and decreased with increasing depth from the impact point. Therefore, the peak acceleration of the recorded data can be an important indicator for evaluating the effective depth of the soil compaction. Based on the findings in Fig. 10, the normal distribution function is used herein to represent the distribution of the threshold peaks from the field (Fig. 11). Figure 12 shows the distribution of the mean (µ) and standard deviation (σ) calculated by threshold peak acceleration. It indicates that a larger µ and σ represent a more significant compaction effect (larger soil distortion). Moreover, this finding could be extracted within 1–10 blows and the trend was almost the same for 11–50 blows.

As depicted in Fig. 12a, c, the µ at the depth of 0–6 m in the X-direction and Y-direction were 0.42–0.84 g and 0.30–0.70 g within 1–10 blows, respectively. In addition, the µ at a depth of 6–10 m in the X-direction and Y-direction were 0.06–0.32 g and 0.05–0.18 g within 1–10 blows, respectively. Obviously, the depth of 6 m was approximately the boundary, reflecting that large vibration intensity has stronger compaction effect. Furthermore, the σ of threshold peak accelerations were maintained within the 0.10–0.36 range above the compaction effect reached its limit (6 m) within 1–10 blows (Fig. 12b in X-direction and Fig. 12d in Y-direction). In comparison, there was a clear drop to 0.01–0.12 when the compaction effect limit (6 m) of the ground was reached. Therefore, the decrease in σ is an indication of the recorded acceleration from the inconspicuous soil shear distortion and weak soil densification effect (Appendix I display the detailed values calculated from the measurements recorded by the vertically SAA string). As mentioned above, the effective depth was determined to be approximately 6 m when the energy per blow was 16.8 t-m for impact over 50 blows. This result was consistent with the CPT results obtained after RIC trial test. Moreover, the result is in an agreement with the conclusion of Berry et al.^14^, who stated that the effective depth of compaction can generally be determined in the first few blow counts of the RIC (< 10 blows). With an increase in the blow count, achieving an increase in the effective depth becomes more difficult subjected to incompressible groundwater unless the compaction parameters related to the energy per blow are readjusted (e.g., compaction foot weight and fall height).

**Figure 13:**
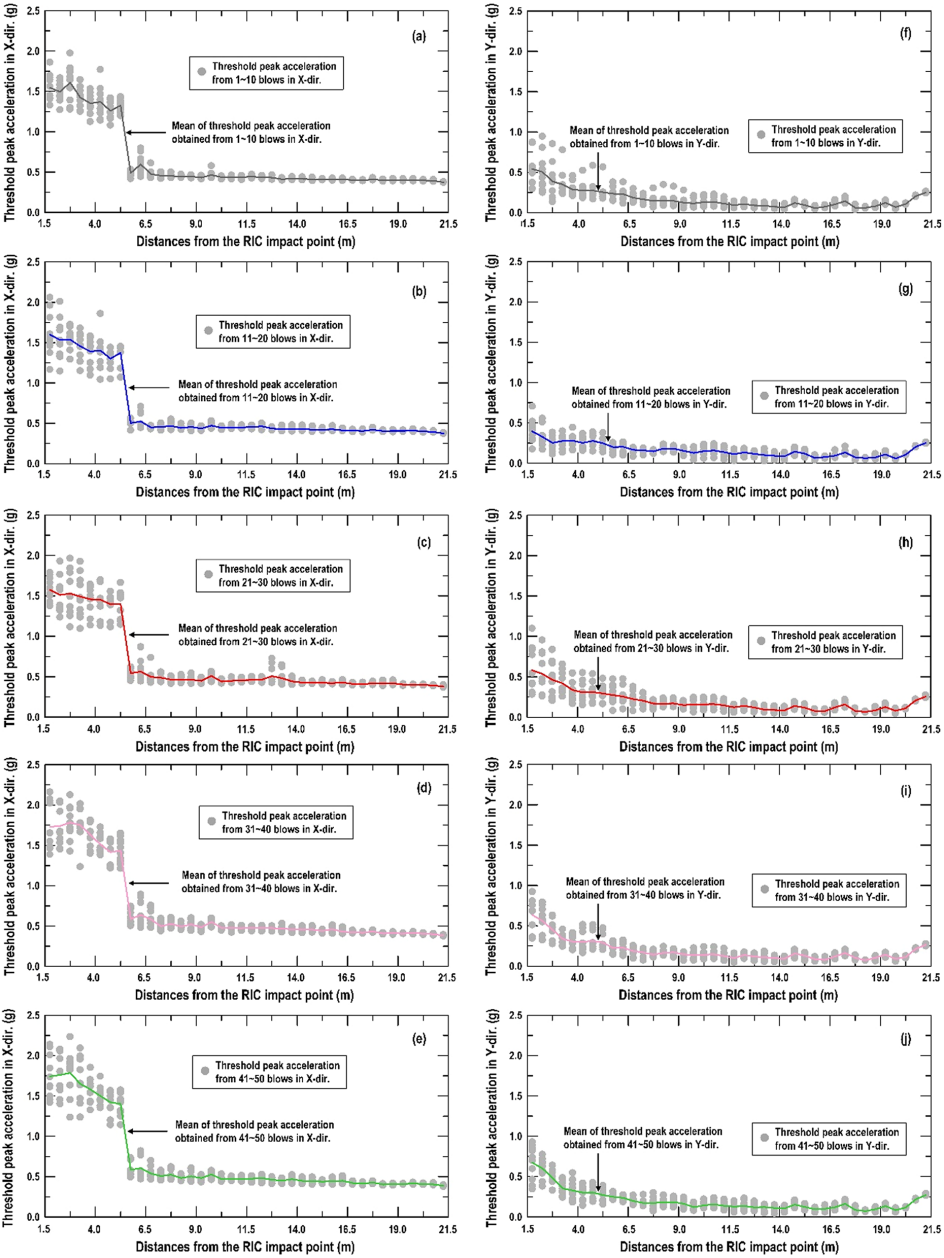
Distributions of the threshold peak acceleration, mean, and standard deviation from the horizontally embedded SAA string during RIC compaction at impact points 1–8: (**a**–**e**) X-direction response during 1–10, 11–20, 21–30, 31–40, and 41–50 blows; (**f**–**j**) Y-direction response during 1–10, 11–20, 21–30, 31–40, and 41–50 blows.

The same data processing was also applied to the recorded data from the horizontally embedded SAA string. Figure 13 displays that both the X-direction and Y-direction threshold peak acceleration distribution extracted from the horizontally SAA string recorded. Same as the trend obtained from vertical SAA string recorded, whatever 1–10 blows, 11–20 blows, 21–30 blows, 31–40 blows, and 41–50 blows, the peak acceleration is maintained to a limit distance and drops down with increasing distance from the compaction point. Therefore, the µ and σ calculated from threshold peak acceleration within 1 to 10 blows can be an important indicator for adjusting the suitable spacing between impact points.

**Figure 14:**
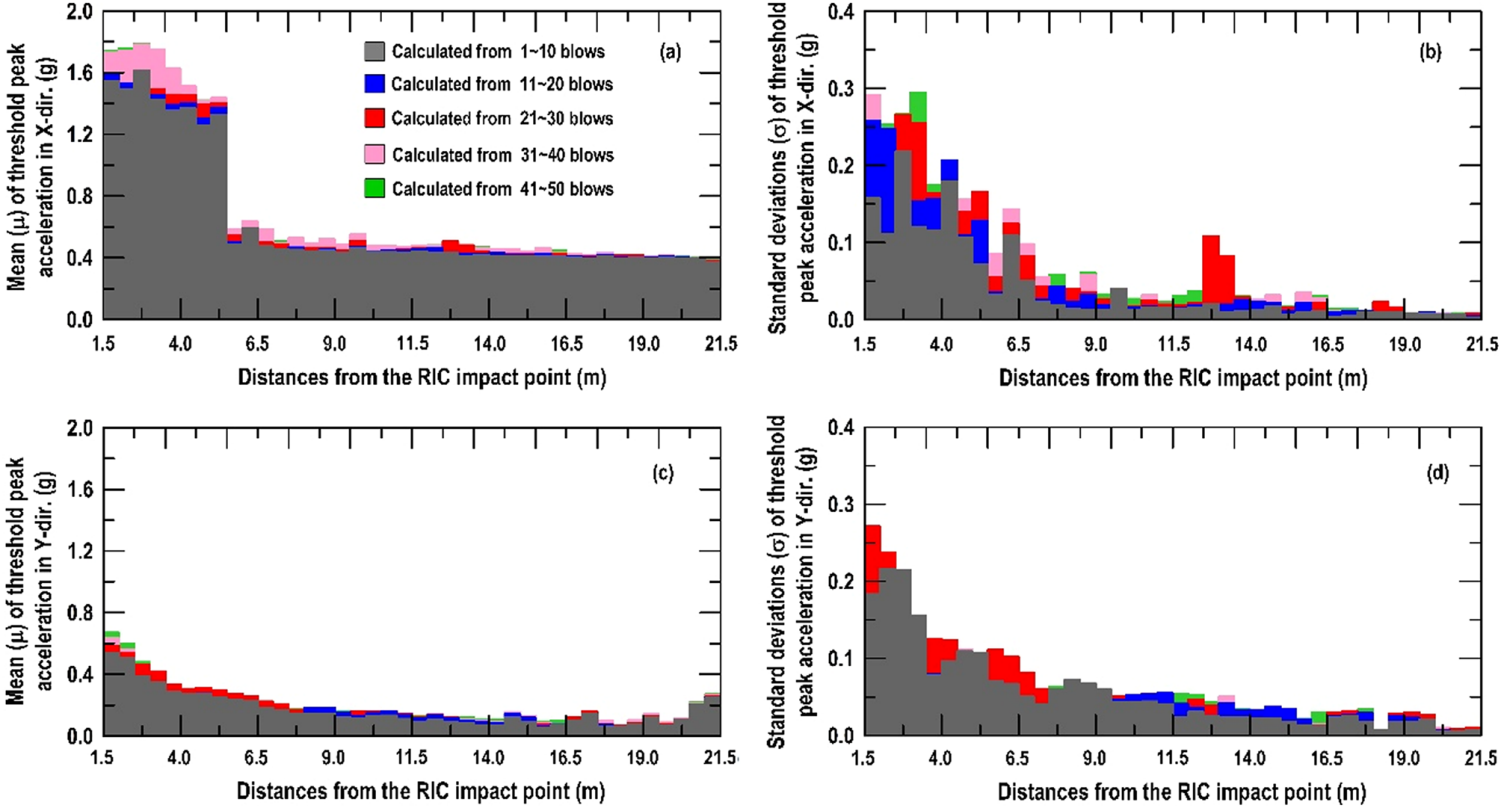
Calculated normal distributions results from the horizontally embedded SAA string recorded: (**a**, **c**) mean of threshold peak acceleration in X-direction and Y-direction; (**b**, **d**) standard deviation of threshold peak acceleration in X-direction and Y-direction.

As depicted in Fig. 14a, c, the µ at the distance of 1.5–5.25 m from the impact point in the X-direction and Y-direction were 1.33–1.61 g and 0.25–0.54 g, respectively. The µ at 5.25–21.5 m from the impact point in the X-direction and Y-direction were 0.39–0.60 g and 0.07–0.27 g, respectively. Unfortunately, because the Y-direction recorded (Fig. 14c) is in parallel to the horizontally embedded SAA string, its response is not obvious in the adjusted of the suitable spacing. Therefore, we need the σ indicator to supply the reliability of impact spacing adjustment. As shown in Fig. 14b (X-direction), d (Y-direction), the σ of threshold peak accelerations were maintained above the 0.10 when the compaction degree reached the distance of 5.25 m within 1–10 blows. In comparison, there was decrease below the value of 0.1 when the compaction degree limit of 5.25 m was reached (Appendix II display the detailed values calculated from the measurements recorded by the horizontally SAA string). Thus, the suitable spacing between impact points was determined to be approximately 5.25 m under energy per blow of 16.8 t-m for 50 blows. The cumulative impact energy only affected the densification in the range of 5.25 m; thus, the spacing between impact points was conservatively designed within this range.

### Spatial analysis of the threshold peak acceleration

**Figure 15:**
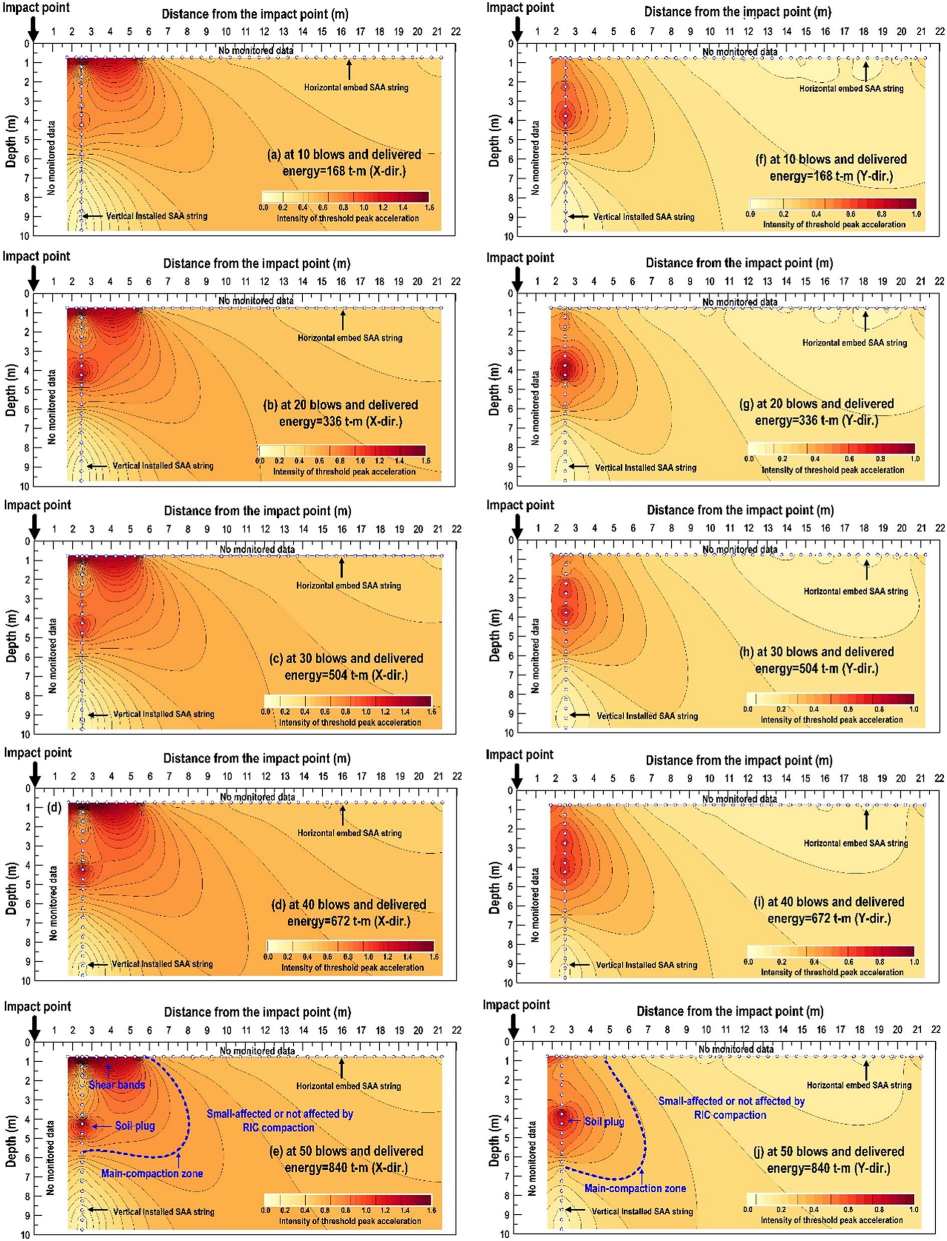
Spatial contour features of the threshold peak acceleration during RIC compaction at impact point 1–8: (**a**–**e**) X-direction contours after 10, 20, 30, 40, and 50 blows; (**f**–**j**) Y-direction contours after 10, 20, 30, 40, and 50 blows.

This study compared the distribution of µ and σ threshold by peak acceleration with the CPT results obtained after RIC. It is demonstrated that used the acceleration caused by the soil shear distortion to evaluate the suitability of the effective zone (combined with effective depth and effective range of compaction degree). The kriged spatial contours were used to analyze the correlations of the threshold peak acceleration with the depth and propagation distance from the RIC impact point for obtaining a clear understanding of the spatial distribution characteristics of the monitored data. Although its reliability depends on the number of measurements recorded in the compaction field, this study adopted the limited data recorded from two vertical and horizontal SAA strings to quickly explain whether the compaction parameters can be used for achieving the required design. By overlapping the threshold peak acceleration from vertical and horizontally SAA strings recorded, Fig. 15 displays the impact points 1–8 and the features of the threshold peak acceleration spatial contours in the X-direction (Fig. 15a–e) and Y-direction (Fig. 15f–j) from the recorded data. The cumulative impact energy for 10, 20, 30, 40, and 50 blows was 168, 336, 504, 672, and 840 t-m, respectively. It illustrates that the spatial contours in the X-direction and Y-direction will change with the cumulative impact energy, but the effective zone of compaction were consistent with the accumulation of impact energy. This finding verified that the compaction effectiveness (effective depth and compaction degree) can be determined within the initial few blow counts (< 10 blows). The key features of the spatial contour map for 50 blows and a cumulative impact energy of 840 t-m (Fig. 15e, j) are explained in the following as an example. A soil plug was formed in the impact process. This plug moved downward to penetrate into deeper soil. The wedging effect caused by the compaction foot induced the development of shear bands that extended from the edges of the impact pit to the ground surface. Under the impact pit, the main compaction zone was formed due to body wave propagation. This zone extended approximately 5 m laterally from the impact point and approximately 6 m vertically from the ground surface. Beyond the main compaction zone, a moderately affected or unaffected zone was formed. This zone had almost no influence on the effective depth and compaction degree. The conclusion is consistent with the idealized spatial profile for RIC at a single impact point reported by Becker^30^ and Jia^32^.

Additionally, it can be found from the spatial contour that the distributions of peak acceleration were different in different directions (X-direction and Y-direction) due to soil anisotropy. However, when the vibration recorded direction is in parallel to the accelerometers (SAA string), the acceleration response is not obvious, and its value may be underestimated, resulting in the invisible development of the shear band. Therefore, if we ignore the influence of soil anisotropy, we infer that the recorded data of X-direction will be more representative than Y-direction.

## Conclusions

RIC is a cost-effective and time-efficient method that is often used for ground improvement to satisfy the bearing capacity, reducing the excessive settlement and resisting liquefaction on the reclaimed land at medium (< 10 m) compaction depth. This study used SAA strings (MEMS accelerators) to monitor the acceleration caused by RIC. Moreover, normal distribution function was used in the data processing to calculate the µ and σ of the threshold peak accelerations from the recorded data, which can be used in compaction operations for evaluating the effective depth and adjusting the suitable spacing between impact points. In contrast to general site investigation tests (e.g., the SPT or CPT), the proposed method cannot provide acceptance values (the SPT-N or q_c_ value) for compaction performance evaluation. However, the trial test results of RIC indicated that the proposed method is suggested for frequent use in individual trial tests of compaction projects to quickly determine the required depth and suitable spacing between compaction points. The following conclusions can be drawn from the findings of this study.

1. The proposed method can be synchronized with the RIC procedure for the real-time monitoring of the acceleration response for various depths and various propagation distances. The statistical analysis of the dispersion degree (µ and σ) of the threshold peak accelerations from recorded data helps effectively evaluating the effective depth and adjusting suitable spacing between impact points of compaction projects.
2. Normal distribution function was employed to obtain the µ and σ of the threshold peak acceleration. These two parameters were used to evaluate the dispersion degree of recorded acceleration for various depths and various propagation distances. A higher vibration intensity (e.g., µ) or dispersion degree (e.g., σ) indicated a stronger shear distortion and soil densification effect, which can be used as indicators of the effective depth and the spacing between impact points.
3. The results of the RIC trial tests revealed that the effective depth and the spacing between impact points could be determined within the initial blow counts (within 10 blows or less). Moreover, no obvious changes were observed as the cumulative energy increased. The achieved performance of a compaction project depends on the site conditions. It can be determined in the early stage of the compaction plan.
4. Spatial contours can be used to establish the correlations of the threshold peak acceleration with the various depths and various propagation distances from the RIC impact point. Moreover, when the number of measurements is adequate, they can indicate the distributions of the effective depth and the spacing between impact points of the vertical and horizontal profiles of the compactor, which can improve the effectiveness of quality control for the compaction project.
5. In addition to SAA string, other useful sensing equipment can be used to measure soil particle acceleration. The amplitude of peak acceleration may change marginally with changes in the accelerometer type and sampling rate. In that, the changes in the accelerometer type and sampling rate did not affect the distribution characteristics of the recorded acceleration at different monitoring locations because the dispersion degree of the calculated peak acceleration represents the compaction performance that can be achieved by the site conditions and setting of compaction parameters.

## Supplementary Information

Supplementary Information.
