# Supplementary material for: Method of estimating the effective zone induced by rapid impact compaction
Source: Sci Rep. 2021 Sep 15;11:18336. doi: 10.1038/s41598-021-97912-1 (PMC8443759; doi:10.1038/s41598-021-97912-1)
Supplement: Supplementary file 1 — Supplementary Information. [file 41598_2021_97912_MOESM1_ESM.docx]

Appendix I Threshold peak acceleration, mean value (μ), and standard deviation (σ) calculated from the measurements recorded by the vertically installed SAA string.

| **X-direction** | **Blow count = 1~10** | | **Blow count = 11~20** | | **Blow count = 21~30** | | **Blow count = 31~40** | | **Blow count = 41~50** | |
| --- | --- | --- | --- | --- | --- | --- | --- | --- | --- | --- |
| Depth (m) | μ (g) | σ | μ (g) | σ | μ (g) | σ | μ (g) | σ | μ (g) | σ |
| 0.25 | 0.4154 | 0.1607 | 0.4067 | 0.0984 | 0.4291 | 0.0820 | 0.6821 | 0.3171 | 0.5741 | 0.1865 |
| 0.75 | 0.6600 | 0.2853 | 0.6076 | 0.1527 | 0.5993 | 0.2403 | 0.6221 | 0.2133 | 0.6351 | 0.1897 |
| 1.25 | 0.5603 | 0.1990 | 0.5604 | 0.2004 | 0.5061 | 0.1665 | 0.4614 | 0.1835 | 0.4483 | 0.1004 |
| 1.75 | 0.4400 | 0.1006 | 0.4566 | 0.1196 | 0.3929 | 0.0776 | 0.4981 | 0.2549 | 0.3390 | 0.1333 |
| 2.25 | 0.7378 | 0.2469 | 0.5983 | 0.1785 | 0.6258 | 0.2886 | 0.6504 | 0.2175 | 0.4035 | 0.1610 |
| 2.75 | 0.7141 | 0.2301 | 0.8005 | 0.2576 | 0.7827 | 0.2232 | 0.7916 | 0.2388 | 0.5096 | 0.2247 |
| 3.25 | 0.6695 | 0.1864 | 0.8452 | 0.2552 | 0.8813 | 0.3493 | 0.8165 | 0.2956 | 0.6373 | 0.2895 |
| 3.75 | 0.7850 | 0.1947 | 1.1162 | 0.2434 | 0.7833 | 0.2916 | 0.5981 | 0.3996 | 0.5385 | 0.3158 |
| 4.25 | 0.8414 | 0.3219 | 1.2759 | 0.2673 | 1.0770 | 0.3747 | 1.2516 | 0.2965 | 1.3674 | 0.1613 |
| 4.75 | 0.6643 | 0.3607 | 0.8150 | 0.5289 | 0.9841 | 0.4887 | 1.0070 | 0.3645 | 1.0220 | 0.3745 |
| 5.25 | 0.6320 | 0.3234 | 0.6630 | 0.3279 | 0.712 | 0.3352 | 0.7495 | 0.3104 | 0.6785 | 0.3611 |
| 5.75 | 0.5222 | 0.2602 | 0.6672 | 0.3366 | 0.6422 | 0.3444 | 0.6035 | 0.3011 | 0.6454 | 0.3417 |
| 6.25 | 0.3180 | 0.1152 | 0.4657 | 0.1163 | 0.4207 | 0.1564 | 0.5717 | 0.2706 | 0.4718 | 0.2378 |
| 6.75 | 0.2025 | 0.0834 | 0.3278 | 0.0682 | 0.2803 | 0.0960 | 0.3584 | 0.2089 | 0.3472 | 0.1417 |
| 7.25 | 0.1655 | 0.0617 | 0.2237 | 0.0532 | 0.2274 | 0.0745 | 0.3439 | 0.1452 | 0.3273 | 0.0730 |
| 7.75 | 0.1750 | 0.0711 | 0.1955 | 0.0418 | 0.2267 | 0.0657 | 0.2736 | 0.0836 | 0.2883 | 0.0543 |
| 8.25 | 0.1049 | 0.0253 | 0.1377 | 0.0267 | 0.1207 | 0.0317 | 0.1986 | 0.0885 | 0.2104 | 0.0862 |
| 8.75 | 0.0808 | 0.0185 | 0.1049 | 0.0227 | 0.1019 | 0.0307 | 0.1524 | 0.0536 | 0.1355 | 0.0289 |
| 9.25 | 0.0806 | 0.0185 | 0.0963 | 0.0176 | 0.0829 | 0.0216 | 0.1172 | 0.0304 | 0.1173 | 0.0266 |
| 9.75 | 0.0659 | 0.0113 | 0.0944 | 0.0170 | 0.0887 | 0.0214 | 0.1271 | 0.0535 | 0.1204 | 0.0383 |
| **Y-direction** | **Blow count = 1~10** | | **Blow count = 11~20** | | **Blow count = 21~30** | | **Blow count = 31~40** | | **Blow count = 41~50** | |
| Depth (m) | μ (g) | σ | μ (g) | σ | μ (g) | σ | μ (g) | σ | μ (g) | σ |
| 0.25 | 0.3677 | 0.1303 | 0.4282 | 0.1862 | 0.3635 | 0.0843 | 0.5689 | 0.2512 | 0.5979 | 0.2334 |
| 0.75 | 0.5824 | 0.2749 | 0.6143 | 0.2526 | 0.5299 | 0.2270 | 0.5581 | 0.2352 | 0.4934 | 0.1126 |
| 1.25 | 0.5059 | 0.2513 | 0.5262 | 0.1036 | 0.4404 | 0.1883 | 0.4662 | 0.1664 | 0.3942 | 0.1080 |
| 1.75 | 0.4639 | 0.1683 | 0.4829 | 0.1967 | 0.4060 | 0.1996 | 0.5696 | 0.1699 | 0.4073 | 0.1352 |
| 2.25 | 0.5994 | 0.1861 | 0.3929 | 0.0779 | 0.6794 | 0.2412 | 0.6545 | 0.2381 | 0.4811 | 0.1626 |
| 2.75 | 0.5268 | 0.2578 | 0.4839 | 0.1669 | 0.6748 | 0.3468 | 0.6894 | 0.2354 | 0.4640 | 0.2094 |
| 3.25 | 0.6074 | 0.2084 | 0.6705 | 0.1824 | 0.5654 | 0.2116 | 0.6031 | 0.2270 | 0.4522 | 0.2173 |
| 3.75 | 0.7028 | 0.3011 | 0.8739 | 0.3026 | 0.7238 | 0.3299 | 0.7035 | 0.3629 | 0.8545 | 0.3473 |
| 4.25 | 0.6213 | 0.1721 | 0.8907 | 0.1528 | 0.6194 | 0.2067 | 0.7034 | 0.1957 | 0.7513 | 0.0912 |
| 4.75 | 0.4298 | 0.2172 | 0.4037 | 0.1521 | 0.4692 | 0.1786 | 0.5916 | 0.2118 | 0.5761 | 0.1624 |
| 5.25 | 0.3978 | 0.2556 | 0.4984 | 0.2681 | 0.5116 | 0.2748 | 0.4933 | 0.2487 | 0.5492 | 0.2011 |
| 5.75 | 0.3016 | 0.1884 | 0.5019 | 0.2429 | 0.4435 | 0.2495 | 0.4513 | 0.2544 | 0.5146 | 0.2891 |
| 6.25 | 0.1763 | 0.0680 | 0.3042 | 0.0790 | 0.2644 | 0.0955 | 0.4319 | 0.2179 | 0.3261 | 0.1516 |
| 6.75 | 0.1163 | 0.0396 | 0.2037 | 0.0612 | 0.1552 | 0.0511 | 0.2418 | 0.1284 | 0.2111 | 0.0843 |
| 7.25 | 0.0882 | 0.0200 | 0.1319 | 0.0210 | 0.1416 | 0.0432 | 0.2438 | 0.1338 | 0.2298 | 0.0836 |
| 7.75 | 0.0777 | 0.0222 | 0.1101 | 0.0321 | 0.1020 | 0.0336 | 0.1467 | 0.0501 | 0.1468 | 0.0422 |
| 8.25 | 0.0578 | 0.0188 | 0.0944 | 0.0273 | 0.0870 | 0.0280 | 0.1296 | 0.0602 | 0.1342 | 0.0390 |
| 8.75 | 0.0457 | 0.0079 | 0.0703 | 0.0192 | 0.0664 | 0.0287 | 0.0953 | 0.0330 | 0.0849 | 0.0348 |
| 9.25 | 0.0485 | 0.0108 | 0.0717 | 0.0447 | 0.0581 | 0.0093 | 0.0790 | 0.0689 | 0.0611 | 0.0247 |
| 9.75 | 0.0469 | 0.0137 | 0.0706 | 0.0178 | 0.0942 | 0.0544 | 0.0816 | 0.0467 | 0.0734 | 0.0251 |

Appendix II Threshold peak acceleration, mean value (μ), and standard deviation (σ) calculated from the measurements recorded by the horizontally embedded SAA string.

| **X-direction** | **Blow count = 1~10** | | **Blow count = 11~20** | | **Blow count = 21~30** | | **Blow count = 31~40** | | **Blow count = 41~50** | |
| --- | --- | --- | --- | --- | --- | --- | --- | --- | --- | --- |
| Distance (m) | μ (g) | σ | μ (g) | σ | μ (g) | σ | μ (g) | σ | μ (g) | σ |
| 1.75 | 1.5467 | 0.1579 | 1.5980 | 0.2585 | 1.5748 | 0.1453 | 1.7303 | 0.2915 | 1.7428 | 0.2558 |
| 2.25 | 1.4939 | 0.1119 | 1.5325 | 0.2472 | 1.5104 | 0.2105 | 1.7401 | 0.2340 | 1.7598 | 0.2532 |
| 2.75 | 1.6107 | 0.2183 | 1.5378 | 0.1215 | 1.5284 | 0.2661 | 1.7833 | 0.1974 | 1.7856 | 0.2669 |
| 3.25 | 1.4248 | 0.1201 | 1.4548 | 0.1543 | 1.4909 | 0.2552 | 1.7512 | 0.2083 | 1.6496 | 0.2951 |
| 3.75 | 1.3523 | 0.1155 | 1.3897 | 0.1564 | 1.4575 | 0.1648 | 1.6293 | 0.1430 | 1.5811 | 0.1746 |
| 4.25 | 1.3689 | 0.1802 | 1.4023 | 0.2068 | 1.4530 | 0.1884 | 1.5123 | 0.1126 | 1.4996 | 0.1186 |
| 4.75 | 1.2576 | 0.1073 | 1.3007 | 0.1093 | 1.3943 | 0.1395 | 1.4152 | 0.156 | 1.4205 | 0.1467 |
| 5.25 | 1.3272 | 0.0720 | 1.3740 | 0.1287 | 1.3996 | 0.1664 | 1.4355 | 0.1449 | 1.4010 | 0.1381 |
| 5.75 | 0.4879 | 0.0326 | 0.5029 | 0.0351 | 0.5431 | 0.0549 | 0.5872 | 0.0857 | 0.5839 | 0.0730 |
| 6.25 | 0.5952 | 0.1094 | 0.5233 | 0.0901 | 0.5647 | 0.1251 | 0.6369 | 0.1438 | 0.6044 | 0.1313 |
| 6.75 | 0.4756 | 0.0504 | 0.4493 | 0.0148 | 0.4985 | 0.0831 | 0.5836 | 0.0984 | 0.5386 | 0.0688 |
| 7.25 | 0.4550 | 0.0233 | 0.4545 | 0.0267 | 0.4881 | 0.0425 | 0.4962 | 0.0554 | 0.5069 | 0.0385 |
| 7.75 | 0.4514 | 0.0192 | 0.4670 | 0.0426 | 0.4650 | 0.0252 | 0.5274 | 0.0433 | 0.5231 | 0.0582 |
| 8.25 | 0.4439 | 0.0138 | 0.4419 | 0.0237 | 0.4628 | 0.0396 | 0.4931 | 0.0287 | 0.4823 | 0.0299 |
| 8.75 | 0.4454 | 0.0134 | 0.4556 | 0.0324 | 0.4633 | 0.0361 | 0.5202 | 0.0582 | 0.5034 | 0.0612 |
| 9.25 | 0.4307 | 0.0132 | 0.4354 | 0.0189 | 0.4496 | 0.0265 | 0.4845 | 0.0253 | 0.4818 | 0.0331 |
| 9.75 | 0.4633 | 0.0401 | 0.4718 | 0.0315 | 0.5102 | 0.0393 | 0.5517 | 0.0299 | 0.5272 | 0.0345 |
| 10.25 | 0.4367 | 0.0130 | 0.4438 | 0.0157 | 0.4406 | 0.0172 | 0.4774 | 0.0173 | 0.4690 | 0.0271 |
| 10.75 | 0.4373 | 0.0163 | 0.4499 | 0.0172 | 0.4493 | 0.0252 | 0.4759 | 0.0318 | 0.4740 | 0.0186 |
| 11.25 | 0.4366 | 0.0158 | 0.4493 | 0.0132 | 0.4573 | 0.0197 | 0.4719 | 0.0204 | 0.4733 | 0.0227 |
| 11.75 | 0.4434 | 0.0140 | 0.4543 | 0.0151 | 0.4603 | 0.0187 | 0.4800 | 0.0184 | 0.4743 | 0.0311 |
| 12.25 | 0.4345 | 0.0155 | 0.4631 | 0.0193 | 0.4642 | 0.0220 | 0.4766 | 0.0196 | 0.4822 | 0.0376 |
| 12.75 | 0.4312 | 0.0202 | 0.4356 | 0.0129 | 0.5077 | 0.1085 | 0.4742 | 0.0172 | 0.4616 | 0.0344 |
| 13.25 | 0.4104 | 0.0100 | 0.4274 | 0.0197 | 0.4825 | 0.0823 | 0.4667 | 0.0294 | 0.4490 | 0.0258 |
| 13.75 | 0.4185 | 0.0108 | 0.4264 | 0.0257 | 0.4425 | 0.0294 | 0.4618 | 0.0179 | 0.4687 | 0.0311 |
| 14.25 | 0.4139 | 0.0121 | 0.4307 | 0.0232 | 0.4276 | 0.0119 | 0.4562 | 0.0265 | 0.4474 | 0.0261 |
| 14.75 | 0.4113 | 0.0169 | 0.4210 | 0.0220 | 0.4248 | 0.0093 | 0.456 | 0.0318 | 0.4433 | 0.0281 |
| 15.25 | 0.4077 | 0.0064 | 0.4205 | 0.0115 | 0.4245 | 0.0159 | 0.4405 | 0.0134 | 0.4379 | 0.0177 |
| 15.75 | 0.4073 | 0.0096 | 0.4257 | 0.0221 | 0.4210 | 0.0100 | 0.4610 | 0.0351 | 0.4431 | 0.0321 |
| 16.25 | 0.4070 | 0.0116 | 0.4128 | 0.0095 | 0.4292 | 0.0218 | 0.4299 | 0.0276 | 0.4483 | 0.0313 |
| 16.75 | 0.4020 | 0.0034 | 0.4089 | 0.0097 | 0.4118 | 0.0088 | 0.4234 | 0.0112 | 0.4205 | 0.0143 |
| 17.25 | 0.3968 | 0.0050 | 0.4041 | 0.0110 | 0.4077 | 0.0110 | 0.4177 | 0.0063 | 0.4137 | 0.0140 |
| 17.75 | 0.4095 | 0.0095 | 0.4206 | 0.0116 | 0.4186 | 0.0111 | 0.4336 | 0.0100 | 0.4267 | 0.0120 |
| 18.25 | 0.3978 | 0.0095 | 0.4036 | 0.0065 | 0.4156 | 0.0233 | 0.4087 | 0.0065 | 0.4026 | 0.0068 |
| 18.75 | 0.3999 | 0.0102 | 0.4057 | 0.0081 | 0.4196 | 0.0163 | 0.4127 | 0.0094 | 0.4055 | 0.007 |
| 19.25 | 0.3987 | 0.0083 | 0.4054 | 0.0074 | 0.4029 | 0.0075 | 0.4074 | 0.0022 | 0.4082 | 0.0043 |
| 19.75 | 0.4026 | 0.0080 | 0.4091 | 0.0099 | 0.4026 | 0.0033 | 0.4162 | 0.0092 | 0.4155 | 0.0086 |
| 20.25 | 0.3958 | 0.0066 | 0.4002 | 0.0074 | 0.3987 | 0.0048 | 0.4063 | 0.0079 | 0.4080 | 0.0074 |
| 20.75 | 0.3930 | 0.0067 | 0.3919 | 0.0068 | 0.3928 | 0.0050 | 0.3986 | 0.0054 | 0.4032 | 0.0080 |
| 21.25 | 0.3729 | 0.0030 | 0.3745 | 0.0046 | 0.3778 | 0.0082 | 0.3837 | 0.0061 | 0.3861 | 0.0064 |
| **Y-direction** | **Blow count = 1~10** | | **Blow count = 11~20** | | **Blow count = 21~30** | | **Blow count = 31~40** | | **Blow count = 41~50** | |
| Distance (m) | μ (g) | σ | μ (g) | σ | μ (g) | σ | μ (g) | σ | μ (g) | σ |
| 1.75 | 0.5412 | 0.1837 | 0.3948 | 0.1417 | 0.5823 | 0.2717 | 0.6338 | 0.2168 | 0.6712 | 0.2026 |
| 2.25 | 0.5049 | 0.2160 | 0.3269 | 0.1130 | 0.5379 | 0.2371 | 0.5602 | 0.1627 | 0.6012 | 0.1170 |
| 2.75 | 0.3869 | 0.2144 | 0.2523 | 0.0801 | 0.4637 | 0.2063 | 0.4507 | 0.1195 | 0.4818 | 0.0981 |
| 3.25 | 0.3494 | 0.1556 | 0.2758 | 0.0770 | 0.4207 | 0.1321 | 0.3383 | 0.0619 | 0.3529 | 0.0812 |
| 3.75 | 0.2858 | 0.0781 | 0.2800 | 0.0797 | 0.3370 | 0.1256 | 0.3044 | 0.0728 | 0.3271 | 0.1116 |
| 4.25 | 0.2744 | 0.0960 | 0.2513 | 0.0700 | 0.3069 | 0.1240 | 0.3007 | 0.1176 | 0.3003 | 0.0878 |
| 4.75 | 0.2743 | 0.1095 | 0.2772 | 0.0837 | 0.3114 | 0.1069 | 0.3119 | 0.1122 | 0.3023 | 0.0825 |
| 5.25 | 0.2521 | 0.1077 | 0.2479 | 0.0725 | 0.2940 | 0.0898 | 0.2914 | 0.1015 | 0.2704 | 0.0749 |
| 5.75 | 0.2350 | 0.0702 | 0.1994 | 0.0487 | 0.2742 | 0.1113 | 0.2255 | 0.0577 | 0.2486 | 0.0352 |
| 6.25 | 0.2306 | 0.0671 | 0.2030 | 0.0552 | 0.2562 | 0.1027 | 0.2266 | 0.0685 | 0.2359 | 0.0290 |
| 6.75 | 0.1841 | 0.0504 | 0.1661 | 0.0327 | 0.2244 | 0.0821 | 0.1870 | 0.0534 | 0.1964 | 0.0313 |
| 7.25 | 0.1647 | 0.0410 | 0.1595 | 0.0286 | 0.2049 | 0.0608 | 0.1700 | 0.0425 | 0.1731 | 0.0381 |
| 7.75 | 0.1444 | 0.0600 | 0.1468 | 0.0185 | 0.1697 | 0.0369 | 0.1417 | 0.0303 | 0.1715 | 0.0638 |
| 8.25 | 0.1476 | 0.0727 | 0.1821 | 0.0273 | 0.1667 | 0.0372 | 0.1649 | 0.0305 | 0.1825 | 0.0535 |
| 8.75 | 0.1462 | 0.0681 | 0.1817 | 0.0238 | 0.1707 | 0.0361 | 0.1713 | 0.0318 | 0.1834 | 0.0473 |
| 9.25 | 0.1237 | 0.0598 | 0.1526 | 0.0263 | 0.1496 | 0.0326 | 0.1453 | 0.0331 | 0.1635 | 0.0536 |
| 9.75 | 0.1143 | 0.0437 | 0.1277 | 0.0464 | 0.1575 | 0.0511 | 0.1345 | 0.0442 | 0.1202 | 0.0401 |
| 10.25 | 0.1304 | 0.0434 | 0.1490 | 0.0528 | 0.1592 | 0.0528 | 0.1409 | 0.0463 | 0.1431 | 0.0378 |
| 10.75 | 0.1339 | 0.0442 | 0.1579 | 0.0535 | 0.1624 | 0.0546 | 0.1454 | 0.0455 | 0.1554 | 0.0384 |
| 11.25 | 0.1107 | 0.0406 | 0.1369 | 0.0553 | 0.1470 | 0.0492 | 0.1239 | 0.0479 | 0.1372 | 0.0451 |
| 11.75 | 0.0929 | 0.0244 | 0.1124 | 0.0412 | 0.1228 | 0.0315 | 0.1002 | 0.0374 | 0.1275 | 0.0542 |
| 12.25 | 0.1057 | 0.0307 | 0.1345 | 0.0358 | 0.1407 | 0.0467 | 0.1377 | 0.0283 | 0.1347 | 0.0529 |
| 12.75 | 0.0900 | 0.0253 | 0.1152 | 0.0258 | 0.1202 | 0.0389 | 0.1189 | 0.0356 | 0.1183 | 0.0435 |
| 13.25 | 0.0862 | 0.0239 | 0.1018 | 0.0420 | 0.0938 | 0.0282 | 0.1096 | 0.0505 | 0.1217 | 0.0349 |
| 13.75 | 0.0721 | 0.0241 | 0.0922 | 0.0328 | 0.0893 | 0.0307 | 0.1012 | 0.0282 | 0.1105 | 0.0343 |
| 14.25 | 0.0666 | 0.0228 | 0.0865 | 0.0336 | 0.0841 | 0.0285 | 0.0923 | 0.0256 | 0.1051 | 0.0308 |
| 14.75 | 0.1199 | 0.0221 | 0.1439 | 0.0369 | 0.1389 | 0.0307 | 0.1559 | 0.0381 | 0.1524 | 0.0363 |
| 15.25 | 0.0936 | 0.0184 | 0.1188 | 0.0347 | 0.1183 | 0.0309 | 0.1294 | 0.0307 | 0.1234 | 0.0275 |
| 15.75 | 0.0558 | 0.0138 | 0.0653 | 0.0211 | 0.0771 | 0.0158 | 0.0867 | 0.0199 | 0.0947 | 0.0214 |
| 16.25 | 0.0729 | 0.0125 | 0.0763 | 0.0073 | 0.0748 | 0.0128 | 0.0786 | 0.0148 | 0.0994 | 0.0296 |
| 16.75 | 0.1031 | 0.0241 | 0.0935 | 0.0254 | 0.1210 | 0.0298 | 0.1194 | 0.0213 | 0.1194 | 0.0282 |
| 17.25 | 0.1435 | 0.0252 | 0.1338 | 0.0267 | 0.1585 | 0.0321 | 0.1630 | 0.0242 | 0.1624 | 0.0301 |
| 17.75 | 0.0611 | 0.0184 | 0.0736 | 0.0292 | 0.0782 | 0.0193 | 0.1021 | 0.0283 | 0.0860 | 0.0337 |
| 18.25 | 0.0601 | 0.0066 | 0.0629 | 0.0071 | 0.0660 | 0.0045 | 0.0722 | 0.0031 | 0.0724 | 0.0077 |
| 18.75 | 0.0800 | 0.0176 | 0.0723 | 0.0249 | 0.0842 | 0.0278 | 0.1038 | 0.0235 | 0.0945 | 0.0259 |
| 19.25 | 0.1225 | 0.0184 | 0.1098 | 0.0232 | 0.1268 | 0.0295 | 0.1417 | 0.0220 | 0.1339 | 0.0241 |
| 19.75 | 0.0680 | 0.0210 | 0.0579 | 0.0148 | 0.0789 | 0.0265 | 0.0886 | 0.0184 | 0.0833 | 0.0179 |
| 20.25 | 0.1072 | 0.0052 | 0.1053 | 0.0065 | 0.1097 | 0.0072 | 0.1136 | 0.0103 | 0.1168 | 0.0073 |
| 20.75 | 0.2039 | 0.0059 | 0.2046 | 0.0053 | 0.2070 | 0.0090 | 0.2147 | 0.0075 | 0.2183 | 0.0059 |
| 21.25 | 0.2493 | 0.0071 | 0.2517 | 0.0058 | 0.2551 | 0.0101 | 0.2640 | 0.0077 | 0.2704 | 0.0076 |
